# Celastrol exerts a neuroprotective effect by directly binding to HMGB1 protein in cerebral ischemia–reperfusion

**DOI:** 10.1186/s12974-021-02216-w

**Published:** 2021-08-09

**Authors:** Dan-Dan Liu, Piao Luo, Liwei Gu, Qian Zhang, Peng Gao, Yongping Zhu, Xiao Chen, Qiuyan Guo, Junzhe Zhang, Nan Ma, Jigang Wang

**Affiliations:** 1 Artemisinin Research Center, and Institute of Chinese Materia Medica, China Academy of Chinese Medical Sciences, Beijing, 100700 China; 2 School of Biopharmacy, China Pharmaceutical University, Nanjing, 210009 China; 3 School of Pharmacy, Jinan University, Guangzhou , 510632 China; 4 Central People’s Hospital of Zhanjiang, Zhanjiang, China; 5 Guangdong Provincial Key Laboratory of New Drug Screening, School of Pharmaceutical Sciences, Southern Medical University, Guangzhou, 510515 China; 6 Department of Physiology, School of Preclinical Medicine, Guangxi Medical University, Nanning, 530021 China; 7 Key Laboratory of Prevention and Treatment of Cardiovascular and Cerebrovascular Diseases, Ministry of Education, Gannan Medical University, Ganzhou, China; 8 Department of Urology, The Second Clinical Medical College of Jinan University, Shenzhen People’s Hospital, 518020 Shenzhen, China

**Keywords:** Celastrol, Chemical proteomics, Target identification, High mobility group protein 1, Cerebral ischemia–reperfusion

## Abstract

**Background:**

Celastrol (cel) was one of the earliest isolated and identified chemical constituents of *Tripterygium wilfordii* Hook. f. Based on a cel probe (cel-p) that maintained the bioactivity of the parent compound, the targets of cel in cerebral ischemia–reperfusion (I/R) injury were comprehensively analyzed by a quantitative chemical proteomics method.

**Methods:**

We constructed an oxygen–glucose deprivation (OGD) model in primary rat cortical neurons and a middle cerebral artery occlusion (MCAO) model in adult rats to detect the direct binding targets of cel in cerebral I/R. By combining various experimental methods, including tandem mass tag (TMT) labeling, mass spectrometry, and cellular thermal shift assay (CETSA), we revealed the targets to which cel directly bound to exert neuroprotective effects.

**Results:**

We found that cel inhibited the proinflammatory activity of high mobility group protein 1 (HMGB1) by directly binding to it and then blocking the binding of HMGB1 to its inflammatory receptors in the microenvironment of ischemia and hypoxia. In addition, cel rescued neurons from OGD injury in vitro and decreased cerebral infarction in vivo by targeting HSP70 and NF-κB p65.

**Conclusion:**

Cel exhibited neuroprotective and anti-inflammatory effects by targeting HSP70 and NF-κB p65 and directly binding to HMGB1 in cerebral I/R injury.

**Supplementary Information:**

The online version contains supplementary material available at 10.1186/s12974-021-02216-w.

## Background

*Tripterygium wilfordii* Hook. f. (*Tw*HF)‐based prescriptions have been widely used for the treatment of autoimmune diseases, tumors, and other diseases in China for centuries [1, 2]. Of the many bioactive constituents isolated from *Tripterygium wilfordii*, celastrol (cel) has attracted close attention for more than 70 years because of its potential medicinal properties [3]. Cel exhibits diverse pharmacological effects over a wide range of disorders, such as cancer, diabetes, obesity [4], and neurodegenerative diseases [3]. Many studies have demonstrated the neuroprotective effects of cel in neurodegenerative diseases through its antioxidant activity and neuroinflammation attenuation capacity [5]. In addition, cel excellently relieved transient global cerebral ischemia and permanent cerebral ischemia induced injury by promoting microglia/macrophage M2 polarization [6], reducing the expression of p-JNK, p–c-Jun and NF-κB [7], and inhibiting the high mobility group box 1 (HMGB1)/NF-κB signaling pathway to exhibit antiinflammatory and antioxidant actions in rats [8]. However, few have focused on determining whether cel has a neuroprotective effect against cerebral ischemia–reperfusion (I/R) injury or identifying its specific binding protein targets.

Neuroinflammatory processes have been implicated in the pathophysiology of multiple stages of cerebral I/R injury, and targeting neuroinflammation has always been an attractive treatment strategy for stroke [9]. Intensive studies of HMGB1, which is currently one of the crucial proinflammatory alarmins of stroke, in inflammation-related diseases have been performed for a number of years. The nonhistone DNA binding protein HMGB1 is primarily located in the cell nucleus and has different biological functions according to the cellular location, binding receptors and redox states. HMGB1 shifts to the cytoplasm and extracellular space by activated immune cells or is passively released by necrotic or damaged cells, and it shows dynamic redox states due to distinct posttranslational modifications [10] and activated inflammatory immune reactions [11]. Outside of the cells, HMGB1 serves as a damage-associated molecular pattern (DAMP) or alarmin to mediate inflammation through receptors, including receptor for advanced glycation endproducts (RAGE) and Toll-like receptors 2 and 4 (TLR2, TLR4) [12]. HMGB1 constitutes three domains: the A box and B box (positively charged) domains and the carboxyl terminus (negatively charged) acidic tail. The three cysteines (Cys) located at positions 23, 45 (A box), and 106 (B box) mainly determine the redox states and physiological functions of HMGB1. Fully reduced HMGB1 isoform presents chemotaxis only by binding to CXCL12 and stimulates immune cell infiltration through the CXCR4 receptor in a collaborative manner. An intramolecular disulfide bond of HMGB1 in Cys 23 and 45 with critical Cys 106 in a reduced state has proinflammatory activity similar to that of cytokines via the TLR4/MD-2 complex, and it induces nuclear NF-κB translocation and produces tumor necrosis factor (TNF) in macrophages. Moreover, the chemotactic and cytokine activities disappear after all Cys are oxidized (sulfonyl HMGB1) [13, 14]. Therefore, the disulfide bond HMGB1 isoform is a biomarker of inflammation, which indicates that blocking the extracellular disulfide bond HMGB1 isoform may be a potential direction for the treatment of inflammation and immune-related diseases, including stroke.

Quantitative chemical proteomics technology based on small molecule compound probes and chemical labeling has been widely used to identify the targets and elucidate the mechanisms of natural and traditional medicines [15], including artemisinin [16], andrographolide [17], curcumin [18], and aspirin [19]. With the help of an activity-based cel probe (cel-p), tandem mass tag (TMT) labeling, liquid chromatography-tandem mass spectrometry (LC–MS/MS), and cellular thermal shift assay (CETSA), we elucidated the neuroprotective mechanisms and targets of cel in cerebral I/R injury and revealed that cel directly bound to HMGB1 to inactivate its cytokine activity and targeted HSP70 and NF-κB to exert anti-inflammatory activity.

## Materials and methods

### Animals

All experiments were carried out for the sake of minimizing the number and suffering of animals. All animal experiment operations meet the requirements of the Beijing Administration Rule of Laboratory Animal and were approved by the Animal Experimental Ethics Review Committee of the Institute of Basic Research for Chinese Medicine, China Academy of Chinese Medical Sciences.

The neonatal Sprague–Dawley rats within 12 h of birth (Vital River Laboratories, Beijing, China) were used for primary cortical neurons isolation. Male adult Sprague–Dawley rats (260–280 g, Vital River Laboratories, Beijing, China) used for middle cerebral artery occlusion (MCAO) experiment were housed in a standard breeding environment without restriction to diet and drinking.

### Reagents

Cell culture reagents: Dulbeccoʼs modified Eagle medium (DMEM), sugar-free DMEM, DMEM/F-12 (1:1), and fetal bovine serum (FBS) were obtained from Corning, USA. Neurobasal-A medium, 50 × B27 supplement, 100 × penicillin–streptomycin (PS), 100 × Glutamax-I, and 0.25% trypsin were purchased from Gibco, USA.

Click chemistry, pull-down, and LC–MS/MS reagents: NaVc, CuSO_4_, TAMRA Azide, and Biotin Azide were obtained from Sigma, USA. High capacity neutravidin agarose resin, sequencing grade modified trypsin, TMT^10^ plex reagent set, Tetraethylammonium bromide (TEAB), and Pierce™ Quantitative Fluorometric Peptide Assay Kit were purchased from Thermo Fisher Scientific, USA. Oasis HLB Extraction Cartridge was obtained from Waters. THPTA was purchased from Click Chemistry Tools.

Other reagents: cel (HPLC > 98%), edaravone (eda) injection (Yangtze River Pharm; China), recombinant human HMGB1 protein (Abcam, USA), cell counting kit-8 (CCK-8; DOJINDO; Japan), and coomassie brilliant blue (CBB; Beyotime; China). The antibodies used in the experiments are shown in Table 1. All other reagents were purchased from Sigma without special instructions.

**Table 1:** Primary antibodies

Primary antibodies	Host	Dilution ratio	Supplier
MAP2	Mouse	1:500 (IF)	Abcam, United States
β-tubulin	Rabbit	1:500 (IF)	Abcam, USA
β-actin	Mouse	1:5000 (WB)	Affinity Biosciences, USA
PCNA	Rabbit	1:3000 (WB)	Proteintech Group, USA
NF-κB p65	Mouse	1:1000 (WB) 1:500 (IF)	Proteintech Group, USA
HSP70	Rabbit	1:1000 (WB) 1:500 (IF)	Abcam, USA
HMGB1	Rabbit	1:1000 (WB) 1:500 (IF)	Abcam, USA
RAGE	Rabbit	1:1000 (WB)	Proteintech Group, USA
TLR4	Rabbit	1:1000 (WB)	Proteintech Group, USA

### Primary rat cortical neuron isolation and RAW 264.7 cell culture

The neonatal Sprague–Dawley rats within 12 h of birth were used for primary neurons isolation as previously established with minor revision [20]. Briefly, the cortices of newborn rats were sterile separated in pre-cooling (4 °C) DMEM/F-12 (1:1). The minced cortex tissue was digested with 0.2 mg/mL DNase I and 2 mg/mL papain, and inactivated by adding 10% volume FBS. The cell suspension was washed twice with DMEM/F-12 (1:1) and resuspended in DMEM/F-12 (1:1) containing 10% FBS and 1 × PS. The cells suspension passed through 300 mesh sieves were seeded on L-polylysine pre-coated well plates or dishes and incubated at 37 °C in an incubator with 5% (v/v) CO_2_. The DMEM/F-12 (1:1) was replaced with complete Neurobasal-A medium replenished with 1 × PS, 1 × Glutamax-I, and 1 × B27 after seeding the cells 4–6 h. The culture medium was changed half every 2–3 days, and all the experiments were carried out on the seventh day unless otherwise stated.

RAW 264.7 cells were cultured in DMEM containing 10% FBS, 1 × PS, and maintained in a 37 °C incubator with 5% (v/v) CO_2_. RAW 264.7 cells grown to 80–90% confluence were digested with 0.25% trypsin and passaged, and TNF-α was tested in cells within 20 passages.

### Oxygen glucose deprivation (OGD) insult

The transient OGD model was constructed to simulate cerebral I/R injury in cultured primary neurons as previously described [21]. Briefly, the Neurobasal-A medium was displaced with deoxygenated, sugar-free DMEM, and the cells were incubated in a hypoxia chamber (STEMCELL Technologies, Canada) filled with 95% N_2_ + 5% CO_2_ for 4 h in a 37 °C incubator and returned to normal culture condition according to the experimental requirement. In contrast, control group cells were cultured in normal culture conditions. After reaching the established time, cell viability was determined by CCK-8 assay or cells were collected for other experiments. For the CCK-8 assay, absorbance was measured using a multimode plate reader (PerkinElmer, USA) at 450 nm.

### Proteome reactivity profiles of primary neurons with cel-p

Labeling profiles of living primary neurons with cel-p were performed in the presence or absence of cel and OGD model by in-gel fluorescence scanning referred to the previous operations [22]. Similarly, different concentrations of cel-p (0–1.6 μM or 0.8 μM, dimethylsulfoxide (DMSO) never exceeded 1% in the final solution) in the absence or presence of competitor (cel, 2 × , 4 × , 6 × , 8 ×) were added into the 6-well plates with or without OGD interfere and incubated for 4 h in a cell incubator. Then supernatant of cell lysate was collected, and the protein concentrations were determined by using the BCA protein assay (Pierce™ BCA protein assay kit). The click chemistry reaction was performed with NaVc (100 mM stock solution, final concentration: 1 mM), THPTA (100 mM stock solution, final concentration: 100 μM), CuSO_4_ (100 mM stock solution, final concentration: 1 mM), and TAMRA Azide (5 mM stock solution, final concentration: 50 μM) in equal amounts (100 μg) of extracted protein for 2 h at room temperature (r.t.). The protein was then precipitated with 1 mL pre-cooling (− 20 °C) acetone and redissolved with 30 μL 1 × SDS loading buffer. The samples (15 μL) were separated with 10% sodium dodecyl sulfate–polyacrylamide gel electrophoresis (SDS-PAGE) gel, and the labeling profiles were visualized by in-gel fluorescence scanning in laser scanner (Azure Sapphire RGBNIR, USA) and then the gels were stained with CBB.

### Cellular imaging

Cellular imaging experiments were carried out as described previously to verify the utility of cel-p for imaging of potential cellular targets [23]. To track the cellular locations of cel-p, living primary neurons were incubated with cel-p 0.8 μM for 0–6 h. The cells were fixed with 4% paraformaldehyde solution for 10 min and 0.2% Triton X-100 permeated for 15 min. Click chemistry reaction was carried out (regents and concentrations as described above) for 2 h and washed thrice to remove excessive agents. The cells were stained with Hoechst for 10 min. DMSO-treated samples were used as control concurrently. Imaging was done with confocal fluorescence microscopy (Leica TCS SP8 SR, Germany).

For colocalization experiments, living primary neurons were incubated with DMSO, 0.8 μM cel-p in the presence or absence of competitor (cel, 8 ×) for 4 h. The cells were fixed, permeated, carried out with click chemistry reaction, and washed as described above. The cells were incubated overnight at 4 °C with anti-HMGB1 antibody and then with secondary fluorescence antibody (goat anti-rabbit, 1:500, Abcam) for 2 h at r.t.. The images were obtained with confocal fluorescence microscopy after staining with Hoechst for 10 min.

### Pull-down/LC–MS/MS and target validation

Pull-down/LC–MS/MS experiments were carried out to identify the interacting cellular targets of cel in primary neurons according to the previous description [24], with the following optimizations. The primary neurons were treated with the cel-p-containing medium (4 μM) in the presence or absence of competitor (cel, 8 ×) for 4 h. Click chemistry reaction was carried out with Biotin Azide (50 mM stock solution, final concentration: 50 μM), NaVc, THPTA, and CuSO_4_ (concentrations as described above) in equal amounts (1 mg) of extracted protein from each sample for 4 h. The proteins were precipitated in pre-cooling (− 20 °C) acetone and redissolved with 0.1% SDS in PBS. The sample supernatants were poured into the washed agarose beads (50 μL). Upon incubation with beads for 2 h at r.t., the beads were washed thrice with 1% SDS, 0.1% SDS, and 6 M urea in order.

For digestion, the beads were resuspended in 500 μL of 6 M urea in PBS and 25 μL of 200 mM DTT in 25 mM NH_4_HCO_3_ buffer. The reaction was incubated at 37 °C for 30 min. Then, the Cys were blocked with 400 mM iodoacetamide (IAA) in 6 M urea and incubated for 45 min in the dark. Sequencing grade modified trypsin (2 μg) was added into each sample and incubated overnight to digest the proteins captured on the beads. The supernatant containing digestive peptides was separated from the mixture by centrifugation and transferred to a new tube. After spin-dry, the dried samples were reconstituted with 100 mM TEAB, and the digestive peptides were labeled with distinct TMT^10^ isobaric peptide tags subsequently on the basis of instructions. Two biological replicates were performed. TMT^10^-126 and TMT^10^-131 label reagents were labeled for negative control samples; TMT^10^-129C and TMT^10^-129 N were labeled for cel-p-treated samples; TMT^10^-130C and TMT^10^-130 N were labeled for cel-p-treated samples in the presence of cel, respectively. After incubation for 2 h, the reaction was quenched with 1 M Tris HCl and the labeled samples were converged to a single new tube. Upon extraction, the samples were desalted by C18 column and then submitted for LC–MS/MS analysis (Thermo Fisher, Orbitrap Fusion Lumos, USA).

### CETSA

For CETSA, experiments were performed according to the previously published protocols with minor modifications [25]. The primary neurons in 100 mm dishes were collected with PBS containing protease inhibitor. The cells were subjected to freezing and thawing cycles in liquid nitrogen and repeated mechanical crushing to obtain cell lysate supernatant by centrifugation. Then equivalent supernatant proteins (1 mg) were treated with either DMSO or cel (20 μM) 1 h with gentle shaking at r.t.. The treated supernatant was divided into 10 equal parts and heated according to designated temperatures. The cooled samples were centrifugated again to obtain supernatant and to perform Western blotting (WB) analysis.

### WB analysis

Supernatants of neuron lysate or rats cerebral cortex tissues lysate were obtained with RIPA lysate in the presence of protease inhibitor. For the cytoplasm and nuclear protein extraction, the nuclear-cytosol extraction kit was used on the basis of instructions. Fully reduced and disulfide bond HMGB1 isoforms were detected in primary neurons and condensed culture supernatant with nonreducing PAGE gel, and samples were collected avoiding the use of reducing agents (*β*-mercaptoethanol or DTT). After OGD- and cel-treatments as detailed above, the culture supernatant was collected at 0 h, 4 h, 8 h, 12 h, 24 h, and 48 h and centrifuged to discard cell debris. Then, the supernatant was concentrated 20 folds with Amicon Ultra-4 50 kDa and Amicon Ultra-4 10 kDa. The protein concentrations were determined by using the BCA protein assay, and the denatured samples were separated with 10%, 12%, or 15% SDS-PAGE gel.

Separated protein samples were transferred to PVDF membranes, blocked in 5% bovine serum albumin (BSA), and incubated overnight at 4 °C with anti-HMGB1, anti-HSP70, anti-NF-κB p65, anti-RAGE, anti-TLR4, PCNA, or β-actin primary antibodies and then with secondary antibodies (goat anti-rabbit, 1:5000; goat anti-mouse, 1:5000) for 2 h at r.t.. Membranes were washed thrice with TBST after incubation. The bands were visualized with enzyme-linked chemiluminescence in the detection system (Azure C400, USA).

### Immunofluorescence (IF) staining

For IF in paraffin sections of rat brain tissue, the paraffin slices were experienced a series of dewaxing and dehydration. The slices were incubated in antigen retrieval for 10 min in 95 °C and permeabilized 15 min in 0.2% Triton X-100. The slices were blocked in 5% BSA for 1 h, incubated with primary antibodies against HMGB1, HSP70, or NF-κB p65 at 4 °C overnight and 2 h with secondary fluorescence antibodies (goat anti-rabbit, 1:500; goat anti-mouse, 1:500, Abcam) in the dark. After 10 min of Hoechst staining, the slices were photographed with a laser scanning confocal microscope.

For IF in primary neurons, the treated cells were washed with PBS, then fixed in 4% paraformaldehyde solution, and permeabilized in 0.2% Triton X-100. The rest procedures were the same with IF processes in rats.

### Expression and purification of HMGB1 A box and B box

Recombinant human HMGB1 A box (residues 1–89) and B box (residues 90–175) were cloned in a modified pET-24d vector (Novagen, Madison, WI) expressing a protein with an N-terminal 6-His tag. The *Escherichia coli* BL21 was transformed with pET24d-HMGB1 A box and pET24d-HMGB1 B box, cultured in LB medium containing 50 g/mL kanamycin at 37 ℃ to an absorbance of 0.8 at 600 nm, and expression was induced with 0.4 mM Isopropyl-D-1-thiogalactopyranoside (IPTG) for 12 h at 16 ℃ before being harvested by centrifugation. Cell pellets were suspended in lysis buffer (20 mM Tris–HCl, pH 8.0, 200 mM NaCl, 1 mM PMSF) and disrupted by sonication. After centrifugation (12,000* g*, 30 min, 4 ℃), the supernatant incorporated His-tagged recombinant A box or B box was applied to a Ni-beads column. The column was washed with 30 mL binding buffer (20 mM Tris–HCl, pH 8.0, 200 mM NaCl and 20 mM or 50 mM Imidazole). A box protein was eluted with elution buffer (20 mM Tris–HCl, pH 8.0, 200 mM NaCl and 200 mM Imidazole). B box protein was eluted with elution buffer (20 mM Tris–HCl, pH 8.0, 200 mM NaCl and 100 mM Imidazole). The samples were exchanged by the buffer containing 20 mM Tris–HCl, pH 8.0, and 200 mM NaCl and concentrated by using centrifugal filter units according to the protocol provided by the manufacturer. The concentration of purified proteins was determined by BCA protein assay reagent kit. The purity and integrity of purified HMGB1 A box and B box were verified by CBB after 15% SDS-PAGE gel separation.

### HMGB1 full-length protein, A box and B box labeling and activity assays

Briefly, recombinant human HMGB1 full-length protein, A box and B box were dissolved or diluted with PBS and reacted with cel, cel-p, IAA, N-Hex-5-ynyl-2-iodoacetamide (IAA-yne), glycyrrhetinic acid (GA), metformin (Met) or DTT as needed for a period of time. Subsequently, the click chemistry reaction was conducted as same as the above fluorescence labeling, and the proteins were separated with 12% (HMGB1 full-length protein) or 15% SDS-PAGE gel (A box and B box). The labeling states were visualized by a fluorescence laser scanner and stained with CBB.

To detect the effect of cel on the proinflammatory activity of recombinant human HMGB1 protein, HMGB1 protein and cel combined HMGB1 protein complex activities were measured by testing the TNF-α content in RAW 264.7 cells after 24 h of treatment. To confirm whether cel blocked the binding of receptors TLR4 and RAGE to B box, the precipitation of B box-cel complex experiment was conducted as described previously with minor revisions [26]. Briefly, B box proteins (100 μg) were first reacted with or without an equivalent amount of cel (equimolar with B box, 10^−2^ μmol) or five-folds amount of cel (5 × 10^−2^ μmol) in Ni-beads column for 1 h. Then 500 μg lysate of primary neurons was added into the Ni-beads column and reacted for 2 h at 4 °C and eluted to precipitate B box or B box-cel complex. The denatured samples were subjected to WB analysis with antibodies against TLR4 and RAGE. The same B box with an equivalent amount of cel elution without incubation with cells lysate was used for TNF-α analysis in RAW 264.7 cells to research whether cel reduced the ability of B box to induce TNF-α secretion.

### Induction of MCAO and neurological defect assessment in rats

By inserting a filament to occlude the right middle cerebral artery (MCA) of male Sprague–Dawley rats (250–280 g), we established the cerebral I/R model in vivo as described previously [27]. Briefly, the rats fasted overnight were anesthetized with a continuous supply of 3% isoflurane/95% oxygen mixture through a rat mask. After cutting a 2-cm long longitudinal incision in the neck, the internal carotid artery (ICA), external carotid artery (ECA) and common carotid artery (CCA) on the right side of the rats were carefully separated with forceps. The ECA was ligated and burned at the distal end of the bifurcation. Temporary occlusion of CCA and ICA with artery clamps, and a 0.36-mm monofilament (2636A4, Beijing Xinong Technology Co., Ltd, China) was inserted into the ICA through a tiny incision in the ECA and advanced approximately 18 mm until slight resistance was felt to block the cerebral blood flow of rats MCA. The rats were anesthetized again 90 min later and the filaments were withdrawn to resume blood stream providing. The rats of the sham group underwent the same steps without inserting the filament. Rats in model (M) + cel group were administered with 1 mg/kg cel, and rats in M + eda group administered with 6 mg/kg eda via caudal vein were used as positive control according to the previously research [6, 28]. For intraperitoneal injection (i.p.), cel was dissolved in 1% DMSO and 0.9% physiological saline in order to yield a concentration of 1 mg/3 mL. The cel and eda were given once at 0 h, 24 h, and 48 h after cerebral I/R, respectively. Four groups included (1) sham, physiological saline i.p.; (2) M, physiological saline i.p.; (3) M + cel 1 mg/kg, i.p.; and (4) M + eda 6 mg/kg, intravenous injection (i.v.).

The neurological deficits of rats were assessed at 24 h, 48 h, and 72 h after reperfusion, respectively, by an experimenter in a blinded experiment. Zea-Longa 5-point scores were used to assess neurologic deficits according to the previous descriptions [27]. Briefly, 0 score indicates no obvious neurological deficit; 1 score indicates left forepaw not fully extended; 2 score indicates the body of the rat circled to the left; 3 score indicates circling around and falling to the left; 4 score indicates unable walk spontaneously and loss of consciousness; and 5 score indicates death. The animals without symptoms of neurological impairment (0 score) or dying (5 score) after surgery were rejected. The animals compatible with requirement were divided into three groups for physiological saline, cel, and eda intervention using a simple random sampling method. Infarct volume was determined by 2, 3, 5-triphenyltetrazolium chloride (TTC) stating and calculated with Image J software as previously [27] 72 h after reperfusion. Briefly, the percentage of the infarct volume = the infarct volumes (white parts)/the whole volume of the cortex. Nissl-stained cells in the rats cerebral cortex were observed at 200 × magnifications with a light microscope to assess Nissl body damage.

### Statistical analysis

Data were presented as the mean ± SEM. Raw data were statistically analyzed with Graph Pad Prism 5.0. The density of WB bands was quantified using Image J software. The data were analyzed using one-way ANOVA. Fisher’s least-significant difference post hoc test was used to test the differences between two groups. The *p* values less than 0.05 were considered as statistically significant.

## Results

### Cel and cel-p showed similar neuroprotective effects in vitro

**Fig. 1:**
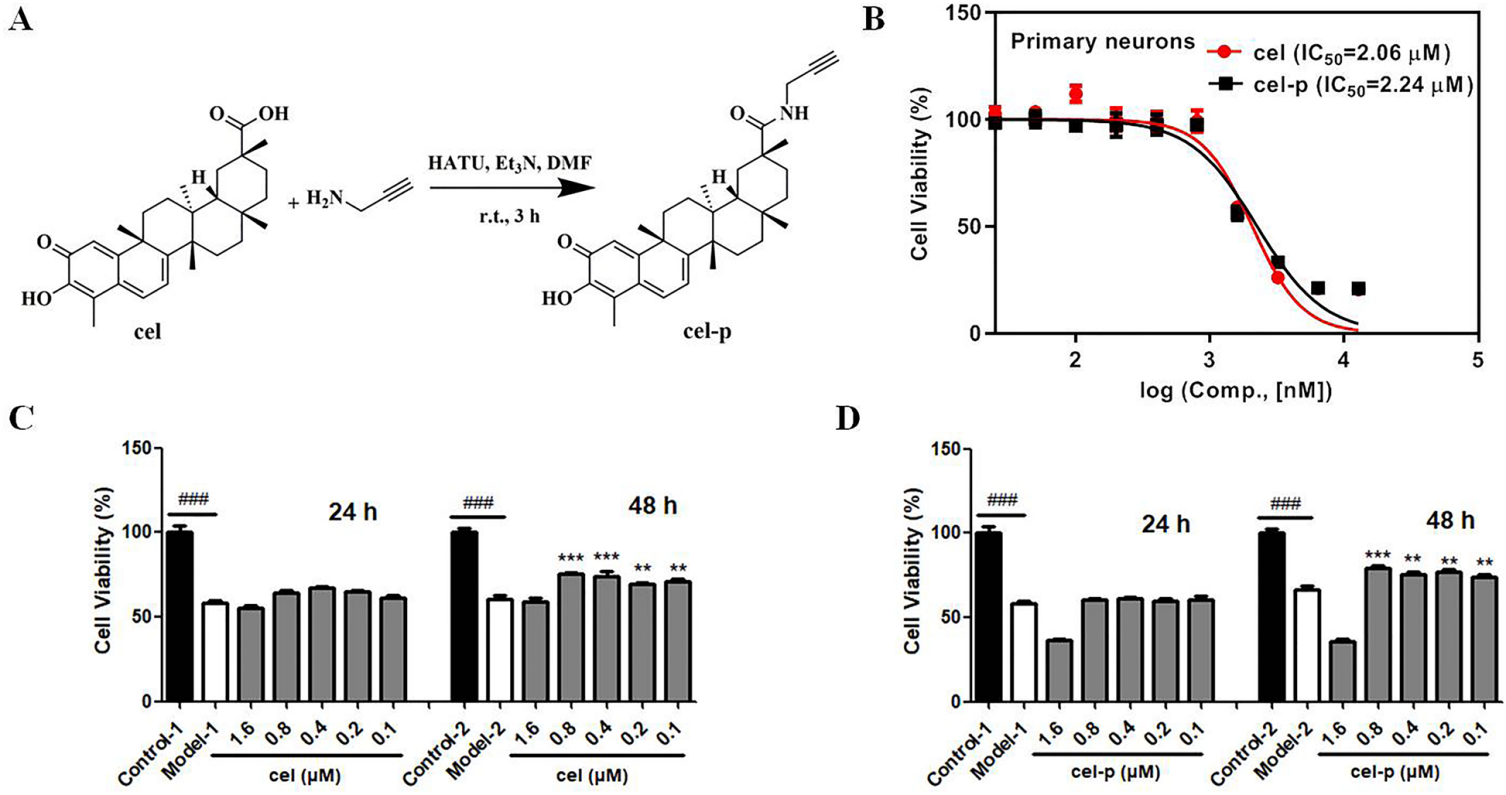
**A** Chemical structure and specific synthesis route of cel-p. **B** IC_50_ values of cel and cel-p against living primary neurons. **C**, **D** Cel and cel-p showed similar neuroprotective effects in an OGD model of primary neurons in vitro. The optimal dose of cel and cel-p was 0.1–0.8 μM, and the optimal administration time was continuous administration for 48 h immediately after OGD. Cel-p closely mimicked the original compound in biological activity. Error bars represent SEM. ^###^*p* < 0.001 *vs.* control group, *p < 0.05, **p < 0.01, ***p < 0.001 *vs.* model group based on a one-way analysis of variance (*n* = 6)

Cel-p was synthesized with an alkynyl handle to the carboxyl terminus of cel (Fig. 1A). We first detected the cytotoxicity of cel and cel-p in living primary neurons. As shown in Fig. 1B, the half maximal inhibitory concentration (IC_50_) results suggested that cel-p closely mimicked the original compound in biological activity (cel IC_50_ = 2.06 ± 0.52 μM, cel-p IC_50_ = 2.24 ± 0.59 μM). For the cell viability assay, primary neurons were incubated in 96-well plates for 7 days and then cultured in an experienced OGD model to evaluate the neuroprotective effect of cel and cel-p. As shown in Fig. 1C, cel exhibited an obvious neuroprotective effect in the OGD model of primary neurons. The optimal dose of cel was 0.1–0.8 μM, and the optimal administration time was continuous administration for 48 h immediately after OGD. Cel-p showed a similar neuroprotective effect as cel (Fig. 1D). Therefore, cel retained its biological activity after introducing biorthogonal reaction groups.

### Cel significantly decreased pathological changes in MCAO rats

**Fig. 2:**
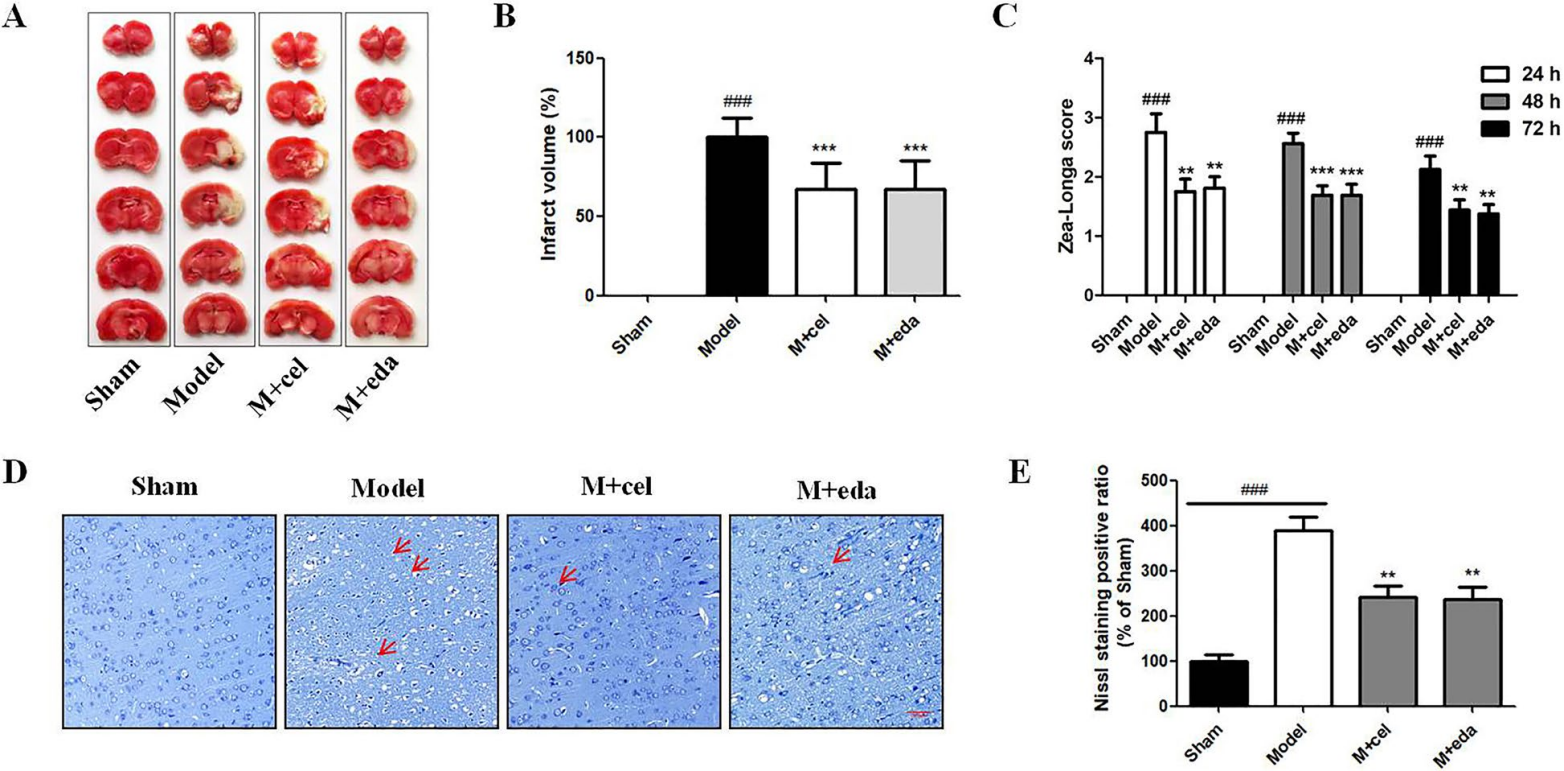
Cel significantly reduced the volume of cerebral infarction, improved the behavior indexes, and decreased Nissl staining injury of MCAO rats. **A** Representative brain slices stained by TTC. **B** Quantitative evaluation of infarct volume. **C** Zea-Longa score results indicated that cel and eda injection improved the behavior indexes of MCAO rats at 24 h, 48 h, and 72 h. **D**, **E** Compared with the sham group, extensive neuronal cell loss, nuclear shrinkage, and dark staining (at the red arrowhead) were visualized in the cortex of the model group. Administration of cel or eda markedly reduced these pathological changes. Error bars represent SEM. ^###^*p* < 0.001 *vs.* sham group, *p < 0.05, **p < 0.01, ***p < 0.001 *vs.* model group based on a one-way analysis of variance was used (*n* = 8, Nissl staining *n* = 3). Scale bar = 200 μm

Previous studies indicated that cel reduced the neurological deficits, brain water content, and infarct volume in a rat permanent cerebral ischemia model [6, 7]. Therefore, we mainly evaluated whether cel exhibited a neuroprotective effect against rats cerebral I/R injury. The TTC staining and quantitative analysis results indicated that cel and eda significantly decreased the infarct volume (Fig. 2A, B, p < 0.001) compared with the model group. According to the Zea-Longa score results, cel and eda notably improved the behavior indexes (Fig. 2C, p < 0.01) at 24 h, 48 h, and 72 h, while significant improvements were not observed in the neurological function of the model group. Moreover, the cel and eda treatments reduced the pathological changes in the cortex of MCAO rats compared with the model group, as shown by Nissl staining (Fig. 2D, E). Therefore, the neuroprotective effect of cel appeared to be similar to eda in vivo.

### Cel-p possessed high bioconjugation efficiency

**Fig. 3:**
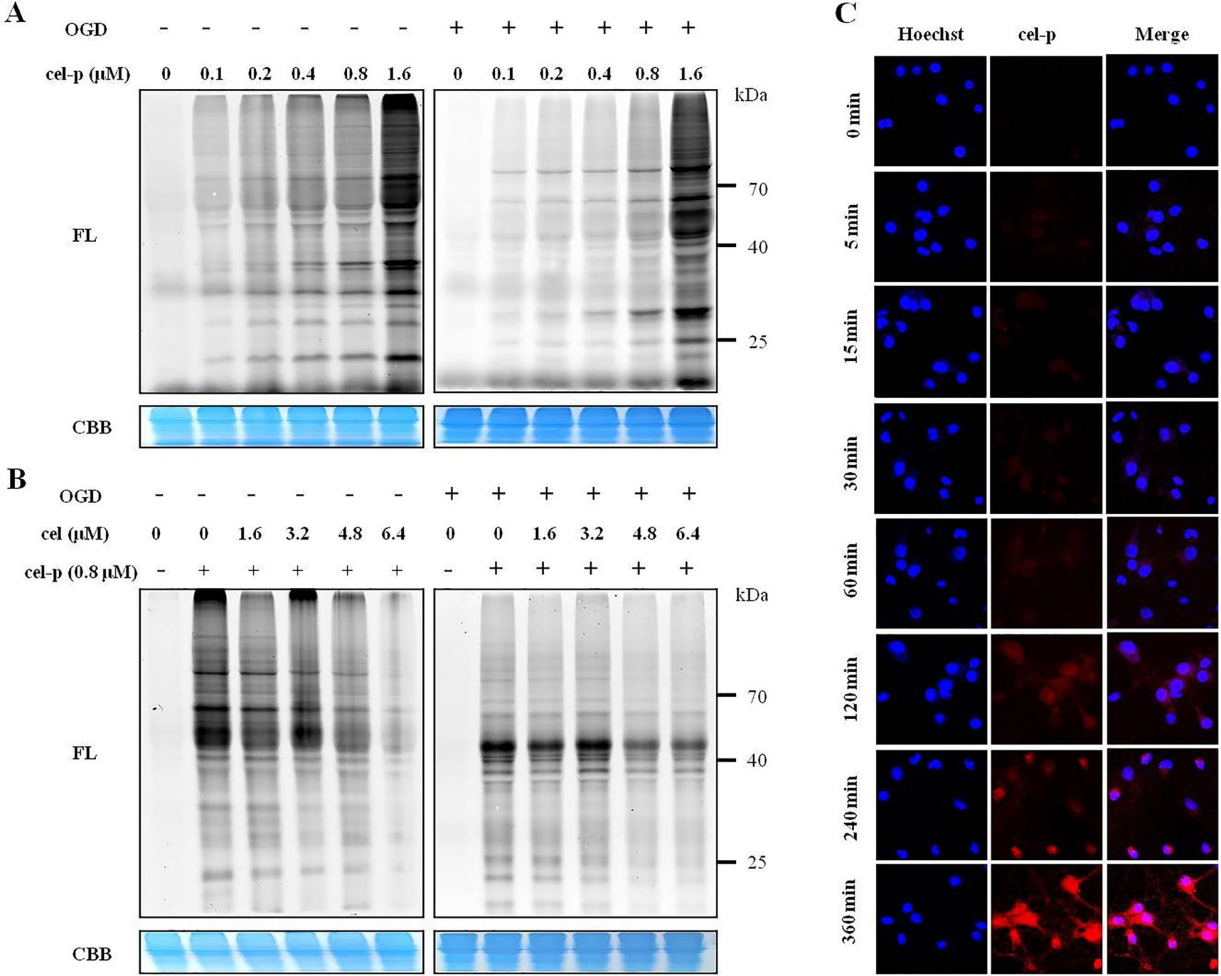
Labeling profiles of cel-p in living primary neurons. **A** Concentration-dependent (0–1.6 μM) labeling profiles of cel-p in normal and OGD injury primary neurons. **B** Competitive labeling profiles of cel-p (0.8 μM) in normal and OGD injury primary neurons in the presence of cel (2 × , 4 × , 6 × , 8 ×). **C** Cel-p mainly localized in the cell cytoplasm within 2 h and gradually entered the cell nucleus after 2 h. FL, in-gel fluorescence scanning; CBB, coomassie brilliant blue gel

We evaluated the labeling profiles of cel-p in living primary neurons. As shown in Fig. 3A, cel-p showed strong labeling efficiency and concentration-dependent labeling of living primary neurons and produced obviously visible bands at probe concentration as low as 0.8 μM after 4 h of incubation. As shown in Fig. 3B, the labeling profiles of cel-p (0.8 μM) became weaker in the presence of the cel competitor (1.6–6.4 μM), suggesting that cel-p bound similar intracellular targets to cel. Cellular imaging experiments with click chemistry reactions were performed to study the cellular locations of cel-p in living cells. As exhibited in Fig. 3C, cel-p mainly localized in the cytoplasm within 2 h and then gradually entered the nucleus, indicating that cel-p was able to label cytoplasm and nuclear proteins after 4 h of labeling. These data demonstrated that cel-p possessed high bioconjugation efficiency under in situ conditions and was a suitable substitute for cel for subsequent chemical proteomics procedures.

### Cel directly targeted HMBG1 and did not affect the expression of HMGB1

**Fig. 4:**
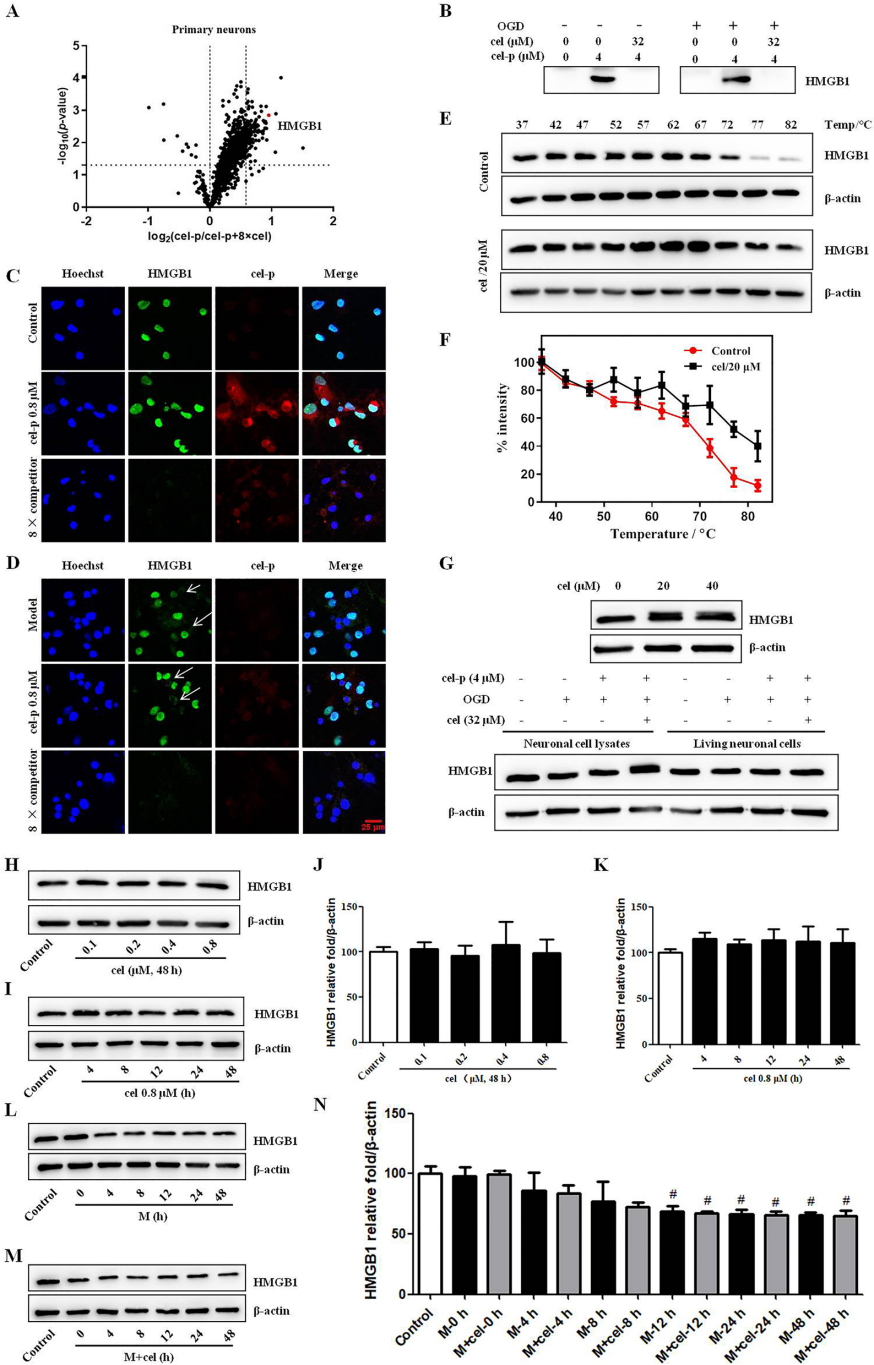
**A** Quantitative mass spectrometry-based profiling of cel-p (4 μM) in the presence of excess cel (8 × , 32 μM). HMGB1 protein had a high degree of credibility. **B**–**D** Target validation of HMGB1 by pull-down, WB, and IF assays in living primary neurons under normal or OGD conditions verified that 8 × cel could completely compete the binding of cel-p to HMGB1 protein. **E**, **F** CETSA results demonstrated that cel directly bound to HMGB1 protein to decrease protein degradation with increasing temperature. **G** High concentrations of cel or cel-p did not affect the expression of HMGB1 in primary neurons lysate or living neuronal cells within 5 h. **H**–**K** Cel had no effect on HMGB1 expression in normal primary neurons within 48 h at 0.1–0.8 μM. **L**–**N** HMGB1 levels were decreasing with time in the M and M + cel groups. Error bars represent SEM. ^#^*p* < 0.05 *vs.* control group based on a one-way analysis of variance was used (*n* = 6). Scale bar = 25 μm

Next, we proceeded to identify the cellular targets of cel by quantitative chemical proteomics technology. Proteins with an enrichment ratio *R*_(cel-p/cel-p + 8 × cel)_ > 1.5 and *p* < 0.05 were set as significant hits, and the protein information labeled by cel-p under this standard was analyzed. The identified protein hits were systematically analyzed and displayed by corresponding volcano plots after cel-p (4 μM) with or without cel (8 × , 32 μM) treatment for 4 h. A total of 1405 proteins were identified by the cel-p target recognition experiment, and 120 of these proteins were highly reliable. A complete list of identified proteins is provided in Table S1. According to these criteria, HMGB1 was identified as one of the direct binding proteins of cel. This identification has fairly high credibility because HMGB1 is currently one of the crucial proinflammatory alarmins of stroke (Fig. 4A). Primary neurons pull-down, WB, and IF assays verified that cel (8 × , 32 μM or 6.4 μM) could completely compete for the binding of cel-p to HMGB1, further demonstrating that HMGB1 was a direct binding protein of cel (Fig. 4B–D). The CETSA results also proved the direct binding of cel and HMGB1 to decrease HMGB1 protein degradation with increasing temperature compared with the control group (Fig. 4E, F).

As shown in Fig. 4C, D, the HMGB1 protein in the cel-treated (8 × , 6.4 μM) group was almost invisible. To confirm whether high concentrations of cel or cel-p affected the expression of HMGB1 in primary neurons, we treated normal living primary neurons with high concentrations of cel (20 μM and 40 μM) for 5 h to evaluate its effect on HMGB1 expression by WB. In the OGD model, we also examined the effect of cel-p (4 μM) in the presence or absence of cel (8 × , 32 μM) on HMGB1 expression in neuronal cell lysates or living neuronal cells. As shown in Fig. 4G, high concentrations of cel or cel-p did not decrease the expression of HMGB1. We speculated that the mechanism of action was the occupation of the binding sites of HMGB1 by high concentrations of cel, which led to the failure of the HMGB1 protein to bind to the HMGB1 antibody, rather than the degradation of HMGB1 by high concentrations of cel or cel-p. In addition, we confirmed that cel had no effect on HMGB1 expression in normal primary neurons at a certain dose and time range (Fig. 4H–K). Unexpectedly, we did not observe a time-dependent increase of HMGB1 expression in the OGD model group. In contrast, the expression of HMGB1 in the model and M + cel groups decreased in a time-dependent manner (Fig. 4L–N). In conclusion, cel directly targeted HMGB1 and did not affect the expression of HMGB1.

### Cel exerted a neuroprotective effect through HSP70 and NF-κB p65

**Fig. 5:**
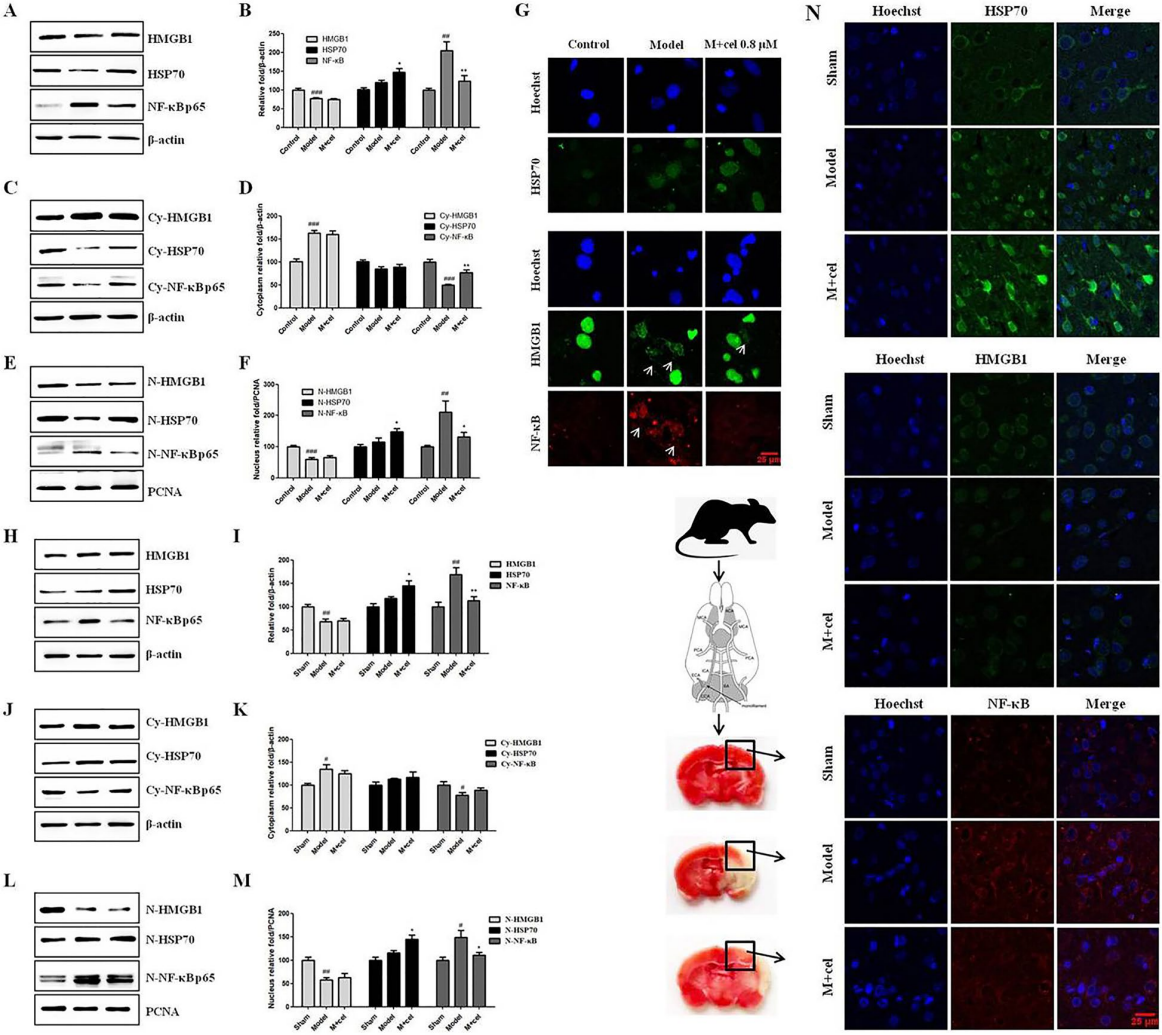
**A**–**J** Changes of HMGB1, HSP70, and NF-κB p65 levels affected by cel under the OGD model were observed at 48 h by WB and IF in vitro. **H**–**N** Changes in HMGB1, HSP70, and NF-κB p65 levels were observed in the rat cerebral cortex by WB and rat cerebral cortex slices by IF. Error bars represent SEM. ^###^*p* < 0.001 *vs.* control group (in vitro) or sham group (in vivo), *p < 0.05, **p < 0.01, ***p < 0.001 *vs.* model group based on a one-way analysis of variance was used (n = 4). Scale bar = 25 μm

As mentioned above, we did not find that cel affected the expression of HMGB1 in primary neurons with or without OGD insult. Previous studies showed that cel induced the HSP70 response and suppressed NF-κB activation to inhibit inflammatory responses and regulate the innate immune response [29–31]. Therefore, we established an OGD model in vitro and a MCAO model in vivo to mimic cerebral I/R injury and tested whether cel affected the distribution changes of HMGB1 in the cytoplasm and nucleus and the expression changes of HSP70 and NF-κB p65 proteins. We found that the expression of HMGB1 in the cytoplasm of the model and M + cel groups significantly increased while the expression of HMGB1 in the nucleus obviously decreased (Fig. 5A–F). In summary, the overall HMGB1 expression was barely affected by cel, which had a limited effect on the distribution of HMGB1 in the cytoplasm and nucleus 48 h after OGD injury. In contrast, cel significantly increased the overall and nuclear expression of HSP70 and decreased the overall and nuclear expression of NF-κB p65 (Fig. 5A–F), which was consistent with the results of previous studies [29–31]. The IF results were in line with the WB results in vitro (Fig. 5J). Similar WB and IF results were also observed in the MCAO rats (Fig. 5H–N). Compared with the model group, cel (1 mg/kg) remarkably increased the expression of HSP70, downregulated the expression of NF-κB p65, and had no effect on the expression of HMGB1 (Fig. 5H–N) in the rats MCAO model. On the whole, cel exerted a neuroprotective effect through increasing the expression of HSP70 and decreasing the expression of NF-κB p65.

### Cel did not affect the secretion and redox states of HMGB1

**Fig. 6:**
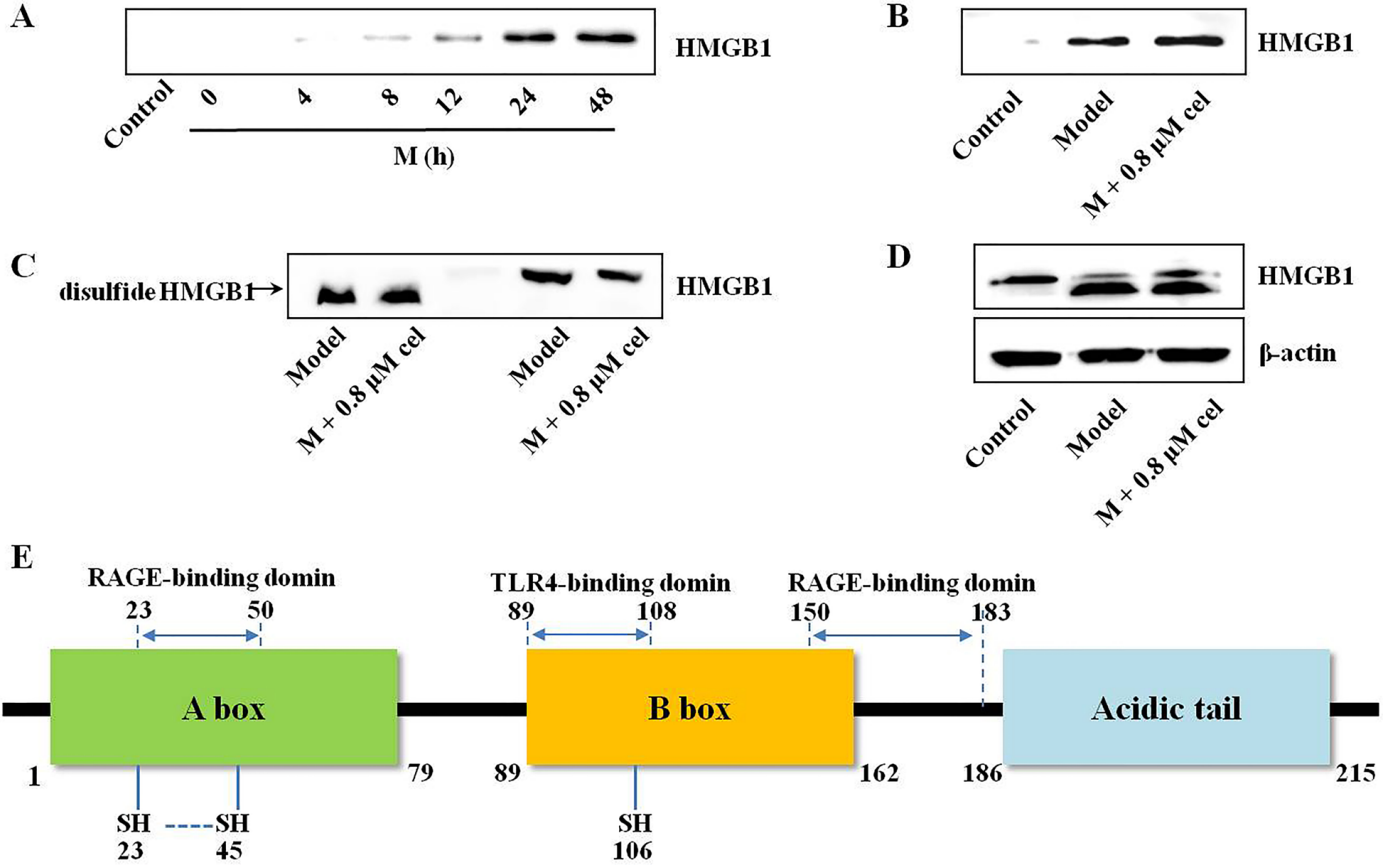
**A** Secreted HMGB1 in the OGD model group supernatant medium evolved over time and reached a peak at 24 h. **B** Cel did not affect the level of secreted HMGB1 in supernatant medium 48 h after OGD injury. The disulfide form (not the fully reduced) HMGB1 isoform occupied the vast majority of HMGB1 isoforms in the OGD supernatant (**C**) and neuron cells (**D**), and cel did not affect the redox states of HMGB1. **E** Structure and function of HMGB1, a 25-kDa protein consisting of 214 amino acids

HMGB1 in the concentrated supernatant of the primary neurons OGD model group increased gradually in a time-dependent manner and reached a peak at 24 h (Fig. 6A). The secreted HMGB1 in the model and M + cel (0.8 μM) groups at 48 h were almost the same (Fig. 6B), which indicated that cel did not affect the secretion of HMGB1 after OGD injury. HMGB1 in the concentrated supernatant was actively secreted in response to OGD injury at 48 h. Cel hardly affected the redox states of HMGB1, which presented as the disulfide bond HMGB1 isoform showed a faster mobility in nondenaturing page gel and cel did not affect its mobility (Fig. 6C). In addition, the disulfide bond but not the fully reduced HMGB1 isoform occupied the vast majority of the model and M + cel groups in primary neurons injured by OGD compared with the control group (Fig. 6D). According to the present results, cel did not affect the secretion and expression of HMGB1 protein or affect the redox states of HMGB1. As shown in Fig. 6E, HMGB1 includes two DNA binding domains (A, B box) and an acidic C-terminal tail. Three Cys residues (Cys 23, 45 and 106) in the A and B boxes mainly determined the redox states of HMGB1. The disulfide bond between Cys 23 and Cys 45 and reduced Cys 106 is indispensable for the binding of HMGB1 to TLR4 and cytokine-inducing activity [10]. Hence, we focused on whether cel bound to HMGB1 to weaken its cytokine activity.

### Cel directly bound to HMGB1, HMGB1 A box, and B box

**Fig. 7:**
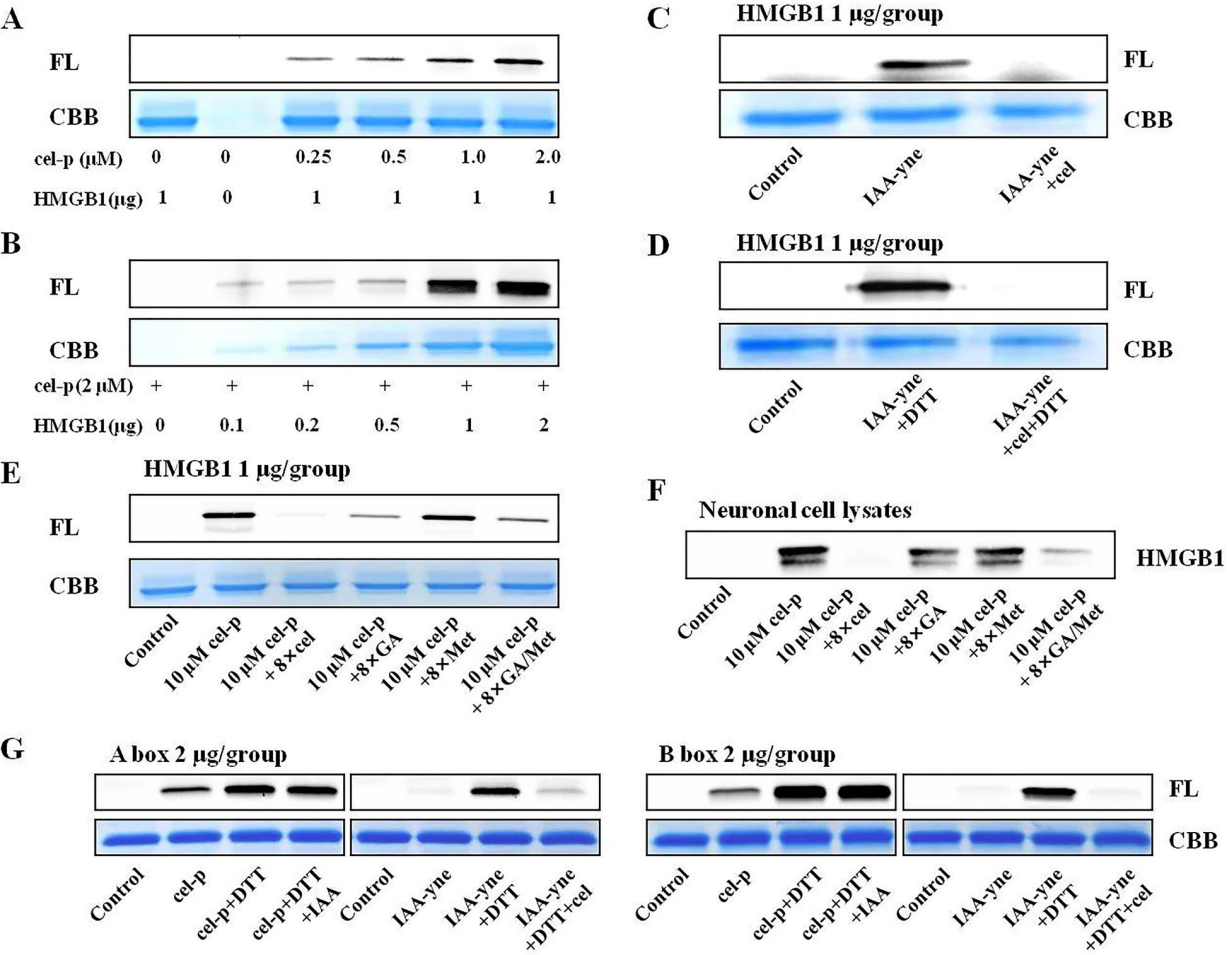
**A**, **B** Cel exhibited strong combining ability with HMGB1 protein in a concentration-dependent manner. **C**, **D** Cel almost completely competed for the binding of IAA-yne with HMGB1, and DTT reduced HMGB1. **E**, **F** The binding ability of cel to the HMGB1 protein was stronger than that of GA and Met. **G** Cel could bind to other sites of HMGB1 in addition to Cys 23, 45 and 106

Cel exhibited strong combining ability with recombinant human HMGB1 protein in a concentration-dependent manner (Fig. 7A, B). Cel almost completely competed for the binding of IAA-yne to HMGB1 or DTT reduced HMGB1 (Fig. 7C, D), which indicated that cel could occupy Cys 106 of disulfide bond HMGB1 isoform or Cys 23, 45, 106 of fully reduced HMGB1 isoform. This result was consistent with previous studies showing that cel could exert cellular effects by forming covalent adducts with Cys residues of proteins [32–34]. The binding ability of cel to the HMGB1 protein was stronger than that of GA and Met, which are recognized HMGB1 inhibitors that bind to the A and B boxes and the C-terminal acidic tail of HMGB1, respectively (Fig. 7E, F). Cel also almost completely blocked the binding of IAA-yne to the Cys group of recombinant human HMGB1 A and B boxes. The binding of cel-p to the A and B boxes could not be blocked by IAA (Fig. 7G), which indicated that cel could bind to other sites of HMGB1 in addition to Cys 23, 45 and 106.

### Cel remarkably blocked the cytokine activity of HMGB1 and B box

**Fig. 8:**
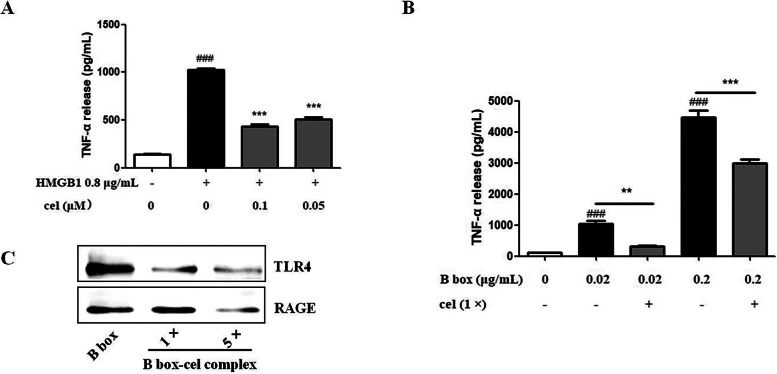
**A** Cel significantly inhibited the secretion of TNF-α induced by HMGB1 in RAW 264.7 cells. **B** Cel obviously decreased the secretion of TNF-α induced by the B box in RAW 264.7 cells. **C** Cel remarkably blocked the binding of TLR4 and RAGE receptors to the B box. ^###^*p* < 0.001 *vs.* control group, **p < 0.01, ***p < 0.001 *vs.* HMGB1 or B box group based on a one-way analysis of variance (*n* = 3)

Cel did not affect the expression or redox states of HMGB1 with or without OGD injury. In addition, previous research indicates that only disulfide bond HMGB1 isoform possesses cytokine activity [10]. Therefore, we mainly explored whether cel influenced the disulfide bond HMGB1 isoform induced increasing of TNF-α in RAW 264.7 cells. According to the description of the manual, the 50% effective concentration (EC_50_) of HMGB1 for stimulating RAW 264.7 cells TNF-α production was 0.7855–0.8342 μg/mL. Therefore, we chose 0.8 μg/mL HMGB1 to induce the secretion of TNF-α in RAW 264.7 cells. As shown in Fig. 8A, 0.8 μg/mL of HMGB1 increased TNF-α secretion more obviously than the control group in RAW 264.7 cells. In contrast, 0.1 μM or 0.05 μM cel + 0.8 μg/mL HMGB1 apparently decreased TNF-α secretion. Recombinant human HMGB1 B box (0.02 μg/mL or 0.2 μg/mL) increased TNF-α secretion more obviously than the control group, and cel (1 × , 10^–6^ μmol or 10^−5^ μmol, equimolar with B box) decreased TNF-α secretion apparently in RAW 264.7 cells (Fig. 8B). According to the published literature, TLR4 is the only receptor of HMGB1 that produces cytokines by binding to the B box Cys 106 [35]. Then, we utilized a recombinant human B box and B box-cel (1 × or 5 × , cel was equimolar with or five times molar of B box) complex to verify that cel obviously blocked the binding of TLR4 and RAGE receptors to the B box (Fig. 8C). In conclusion, cel remarkably blocked the cytokine activity of HMGB1 and B box by directly binding to them to block the combination of inflammatory receptors.

## Discussions

**Fig. 9:**
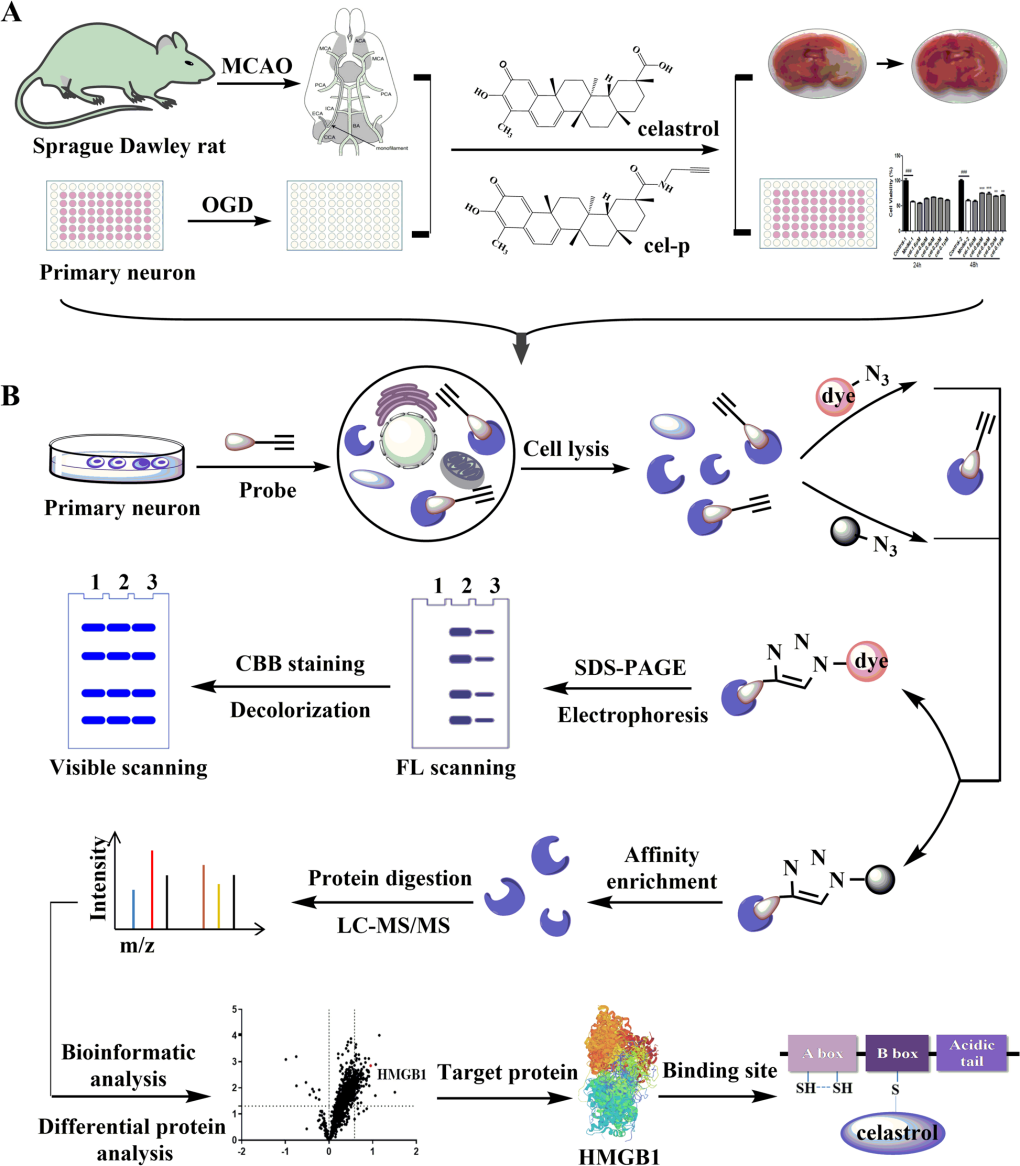
**A** OGD model of primary neurons and MCAO model of adult rats were established to demonstrate that cel had a neuroprotective effect and its modified product (cel-p) was with retained the biological activity. **B** HMGB1 was verified as an important target directly bound by cel based on biorthogonal reaction, TMT labeling, LC–MS/MS, and CETSA. Cel had an anti-inflammatory effect by targeting HSP70 and NF-κB p65 and did not affect the expression levels of HMGB1. Cel blocked the cytokine activity of HMGB1 and B box by directly binding them to disrupt their binding with inflammatory receptors

Our studies demonstrated the following: (1) cel exhibited a neuroprotective effect against cerebral I/R injury in vitro and in vivo; (2) cel directly bound to HMGB1 protein; (3) cel did not affect the expression of HMGB1, although it increased HSP70 and decreased NF-κB p65 expression to exert a neuroprotective effect in vitro and in vivo; (4) cel bound to Cys 106 of the disulfide bond HMGB1 isoform or Cys 23, 45, 106 of the fully reduced HMGB1 isoform; (5) cel scavenged the overproduced TNF-α induced by the disulfide bond HMGB1 isoform and B box; and (6) cel exerted an anti-inflammatory effect by binding to the B box to block the combination of receptors TLR4 and RAGE with the HMGB1 B box. Taken together, our findings suggested that the neuroprotective action of cel for ischemia stroke was partly due to its inhibition of neuroinflammation through upregulating HSP70 and downregulating NF-κB p65 expression and directly binding with HMGB1 protein. The specific experimental process is shown in Fig. 9.

With the maturation of mass spectrometry-based proteomics technology, stable isotope labels have been widely used in research on the biomarkers for diseases and drug targets through quantitative measurements of relative or absolute protein amounts in healthy versus disease states [36]. TMT reagents are commercially available and widely applied isobaric tags that allow multiplexing of up to 10 samples with high-resolution instruments and a range of sample types applicable [37]. TMT isobaric labeling could simultaneously identify and quantify complex protein mixture components with the key workflow of sample denaturation, digestion, isobaric tagging of tryptic peptides, fractionation, mass spectrometric analysis, and data processing [38, 39]. First, we verified that cel retained its biological activity after introducing biorthogonal reaction groups. Cel exhibited a neuroprotective effect against ischemia stroke in vitro and in vivo. With the aid of cel-p, TMT labeling, and LC–MS/MS technologies, we identified 1405 proteins in the cel-p target recognition experiment, and 120 were highly reliable. HMGB1 was identified as a direct binding protein of cel with fairly high credibility (Fig. 4).

CETSA is a label-free biophysical technique for studies of target engagement in cells and tissues based on ligand binding that affects protein stability and cellular studies of protein redox modulations [40]. Generally, many proteins unfold after heating and precipitate rapidly in cells and drug-binding proteins can stabilize proteins and reduce their degradation with increasing temperature compared with untreated cells [41]. CETSA based on immunoassay (such as WB, proximity ligation assays, and mass spectrometry) detection is a hot technique for validating ligand binding of drugs to proteins in lysates, cells, and tissues, and it is based on measuring the protein melting curve changes after different heating steps and quantifying the amount of remaining soluble protein [41, 42]. We investigated whether cel combined with and stabilized HMGB1 in cell lysate samples subjected to temperatures from 37 to 82 °C. The results showed that compared with the DMSO-treated control group, the cel-treated group significantly stabilized HMGB1 and decreased its degradation with increasing temperature (Fig. 4E, F).

HMGB1 is highly expressed in the nucleus of multiple cell types, and the redox states of intracellular and extracellular HMGB1 are dynamic and mainly related to Cys 23, 45, and 106. The disulfide bond HMGB1 isoform (Cys 106 in thiol and Cys 23, 45 form disulfide bond) is required for the TLR4/MD-2 interaction to induce TNF release and NF-κB activation [10]. HMGB1 can be released by passive or active secretion via multiple pathways. Passive release of HMGB1 occurs rapidly during primary necrosis with the fully reduced or disulfide bond isoforms or nuclear retention and passive release during cell apoptosis secondary necrosis with a mainly fully oxidized isoform (sulfonyl HMGB1). Active secretion occurs in the late stage of pyroptosis with posttranslational modification and mainly occurs with the disulfide bond isoform [12]. Previous studies showed that cel significantly suppressed the HMGB1/NF-κB pathway to alleviate inflammatory pain, exhibited a neuroprotective effect in transient global cerebral I/R, and inhibited HMGB1 expression to decrease myocardial I/R injury [8, 43, 44]. In contrast to previous studies, the peak expression of HMGB1 was not detected in the model group and cel did not affect the expression of HMGB1. The results of this experiment may be related to the type of cells we selected. Because mammalian neurons are terminally differentiated, postmitotic cells and isolated primary rat cortical neurons have little ability to divide and proliferate in vitro in the absence of inducers [45]. The WB results for the neuron cells culture medium supernatant demonstrated that primary neurons suffering from OGD injury mainly actively secreted the disulfide bond HMGB1 isoform and the formation of disulfide bond could hardly be prevented by cel (Fig. 6). Plasma HMGB1 rapidly increases and acts as a proinflammatory cytokine to activate microglia, aggravate excitotoxicity-induced neuronal death, and aggravate brain injury during the acute damaging phase of ischemia insult [46, 47]. Early HMGB1 translocation and release occurs mostly in injured neurons and acts as a proinflammatory cytokine by interacting with receptors of RAGE, TLR2, and TLR4 [48]. High levels of HMGB1 in the serum and cerebrospinal fluid (CSF) are related to the severity of animal ischemia brain damage. In addition, HMGB1 in the serum of lipopolysaccharide (LPS)-administered MCAO animals was upregulated and mainly associated with the disulfide bond type [49]. Blockade of HMGB1 with antagonists has been verified as an effective treatment strategy for animal stroke models, including GA [50], HMGB1 A box, and anti-HMGB1 monoclonal antibody [51]. Previous studies demonstrated that peripheral disulfide bond HMGB1 isoform produced more obvious pronociceptive activity than all-thiol HMGB1 isoform by activating TLR4 rather than RAGE [52]. Here, we confirmed that cel did not affect the secretion, redox states, or expression of HMGB1 in either normal neurons or OGD-exposed neurons. Apart from binding with Cys, cel also occupied other sites of HMGB1. Considering that only the disulfide bond HMGB1 isoform has cytokine properties, we focused on the effect of cel on disulfide bond HMGB1 isoform. Cys106 is the main binding site of the TLR4 receptor to HMGB1 to exert cytokine activity. In our research, cel directly bound to the HMGB1 A and B boxes and blocked the binding of the HMGB1 B box to its receptors TLR4 and RAGE, which resulted in inflammatory activity loss. Cel disrupted the TNF-α-inducing capacity of HMGB1 and B box in RAW 264.7 cells. Therefore, cel was directly binding to HMGB1 protein to inhibit its inflammatory activity rather than reducing its secretion or changing its redox activity. In addition, cel exerted anti-inflammatory effects in cerebral I/R injury by targeting HSP70 and NF-κB p65.

Although cel and its numerous derivatives exhibit potential therapeutic effects against various diseases, none of them have been approved for clinical use due to their toxic effects, low solubility, and narrow therapeutic dose range [53]. Therefore, identifying a method of resolving the toxicity of cel and improving its efficacy represents the next research direction. In addition, a growing body of evidence supports the idea that inflammation plays different roles in different stages of stroke [54]. HMGB1 shows different activities according to its redox modifications and may play a more complex role in ischemia stroke, which remains to be explored. In addition to cytokine activity, HMGB1 also exerts beneficial effects in axonal regeneration, endothelial activation, angiogenesis, neurovascular repair, and remodeling [11, 55]. Therefore, it is necessary to carefully consider whether to promote or inhibit HMGB1 in different stages of stroke.

## Conclusions

In summary, we performed a proteome-wide investigation of direct cellular protein binding targets of cel in primary rat cortical neurons and identified 120 targets with fairly high credibility through a quantitative chemical proteomics approach. The present study demonstrated the neuroprotective effect of cel against cerebral I/R injury by targeting HSP70 and NF-κB p65 and disrupting the cytokine activity of the disulfide bond HMGB1 isoform in a primary neurons OGD model and an adult rats MCAO model. The findings presented here may provide a potential therapeutic direction for ischemia stroke therapy. By directly binding to the HMGB1 B box, cel blocked the binding of the TLR4 and RAGE receptors with the B box, which promoted anti-inflammatory activity. To the best of our knowledge, this is the first study to evaluate the direct binding of cel to HMGB1. We hope that these data and findings from the present study could provide guidance for the clinical use of cel in the future.

## Supplementary Information

**Additional file 1.** Supplementary Table S1.

## Data Availability

The datasets and materials supporting the conclusions of this article are included within the article.

## Abbreviations

OGD: Oxygen-glucose deprivation
MCAO: Middle cerebral artery occlusion
TMT: Tandem mass tags
CETSA: Cellular thermal shift assay
TEAB: Tetraethylammonium bromide
WB: Western blotting
IF: Immunofluorescence
Cys: Cysteine
HMGB1: High mobility group protein 1
DAMP: Damage-associated molecular pattern
RAGE: Receptor for advanced glycation endproducts
TLR: Toll-like receptor
TNF: Tumor necrosis factor
cel: Celastrol
cel-p: Celastrol probe
eda: Edaravone
LC–MS/MS: Liquid chromatography-tandem mass spectrometry
DMEM: Dulbeccoʼs modified Eagle medium
FBS: Fetal bovine serum
DMSO: Dimethylsulfoxide
r.t.: Room temperature
I/R: Ischemia-reperfusion
i.p.: Intraperitoneal injection
i.v.: Intravenous injection
SDS-PAGE: Sodium dodecyl sulfate–polyacrylamide gel electrophoresis
IAA: Iodoacetamide
IAA-yne: N-Hex-5-ynyl-2-iodoacetamide
LPS: Lipopolysaccharide
CCK-8: Cell counting kit-8
CBB: Coomassie brilliant blue
ICA: Internal carotid artery
ECA: External carotid artery
CCA: Common carotid artery
IC_50_: 50% Inhibiting concentration
EC_50_: 50% Effective concentration
GA: Glycyrrhetinic acid
Met: Metformin
TTC: 2, 3, 5-Triphenyltetrazolium chloride
DTT: Dithiothreitol
CSF: Cerebrospinal fluid
IPTG: Isopropyl-D-1-thiogalactopyranoside
